# Suppression Effect of Astaxanthin on Osteoclast Formation In Vitro and Bone Loss In Vivo

**DOI:** 10.3390/ijms19030912

**Published:** 2018-03-19

**Authors:** Yun-Ho Hwang, Kwang-Jin Kim, Su-Jin Kim, Seul-Ki Mun, Seong-Gyeol Hong, Young-Jin Son, Sung-Tae Yee

**Affiliations:** Department of Pharmacy, Sunchon National University, 255 Jungangno, Suncheon 540-950, Korea; hyh7733@naver.com (Y.-H.H.); mastiffk@naver.com (K.-J.K.); ksz1353@naver.com (S.-J.K.); motomoto1210@naver.com (S.-K.M.); hong9217@naver.com (S.-G.H.); sony@sunchon.ac.kr (Y.-J.S.)

**Keywords:** Astaxanthin (AST), osteoporosis, bone mineral density (BMD), osteoclast, nuclear factor of activated T cells c1 (NFATc1)

## Abstract

Osteoporosis is characterized by a reduction of the bone mineral density (BMD) and microarchitectural deterioration of the bone, which lead to bone fragility and susceptibility to fracture. Astaxanthin (AST) has a variety of biological activities, such as a protective effect against asthma or neuroinflammation, antioxidant effect, and decrease of the osteoclast number in the right mandibles in the periodontitis model. Although treatment with AST is known to have an effect on inflammation, no studies on the effect of AST exposure on bone loss have been performed. Thus, in the present study, we examined the antiosteoporotic effect of AST on bone mass in ovariectomized (OVX) mice and its possible mechanism of action. The administration of AST (5, 10 mg/kg) for 6 weeks suppressed the enhancement of serum calcium, inorganic phosphorus, alkaline phosphatase, total cholesterol, and tartrate-resistant acid phosphatase (TRAP) activity. The bone mineral density (BMD) and bone microarchitecture of the trabecular bone in the tibia and femur were recovered by AST exposure. Moreover, in the in vitro experiment, we demonstrated that AST inhibits osteoclast formation through the expression of the nuclear factor of activated T cells (NFAT) c1, dendritic cell-specific transmembrane protein (DC-STAMP), TRAP, and cathepsin K without any cytotoxic effects on bone marrow-derived macrophages (BMMs). Therefore, we suggest that AST may have therapeutic potential for the treatment of postmenopausal osteoporosis.

## 1. Introduction

Osteoporosis has been widely recognized as a major health issue and is a skeletal disease characterized by the reduction of bone mass and deterioration of the microarchitecture in bone tissue with a consequent increase in bone fragility [1]. The clinical and economic burden of patients with fractures of the hip and femur is high. Effective interventions to reduce the risk of fracture have the potential to yield substantial cost savings [2]. Osteoporosis often occurs in postmenopausal women and is associated with estrogen deficiency; 75% of postmenopausal women suffer from bone loss. Immediately after the menopause, their bone mass decreases by 3–5% per year and about 1.5 million people suffer from osteoporosis related fractures each year [3].

Estrogen deficiency increases osteoclast formation by providing a larger recruited osteoclast progenitor pool. The upregulated formation and activation of osteoclasts increases the resorption area of the trabecular bone surface [4]. Osteoclast differentiation is supported by cells of the osteoblast lineage and this process is facilitated by the stromal cells of the bone marrow. The cytokines required for osteoclast differentiation are the receptor activators of nuclear factor kappa-B ligand (RANKL) and macrophage colony stimulating factor (M-CSF) [5,6]. RANKL is expressed on the osteoblast cells and induces the essential signal for precursor cells to differentiate into osteoclasts. M-CSF secreted by osteoblasts supplies the survival signal to these cells. The binding of RANKL to its receptor RANK in bone marrow-derived monocyte/macrophage precursor cells (BMMs) results in the recruitment of TNF receptor-associated factor 6 (TRAF 6), which is linked to the nuclear factor kappa-light-chain-enhancer of activated B cells (NF-κB) and the Jun N-terminal kinase (JNK) pathway [7]. The nuclear factor of activated T cells (NFAT) c1 induced by activated NF-κB is essential for osteoclast differentiation [8]. Furthermore, dendritic cell-specific transmembrane protein (DC-STAMP) is associated with cell-to-cell fusion and is essential for multinucleation in osteoporosis [9].

Data of the Women’s Health Initiative (WHI) study show excess risks per 10,000 person-years attributable to estrogen plus progestin were seven more coronary heart disease (CHD) events, eight more strokes, eight more PEs, and eight more invasive breast cancers, while absolute risk reductions per 10,000 person-years were six fewer colorectal cancers and five fewer hip fractures [10]. Estrogen replacement therapy (ERT) has been used to treat postmenopausal women and the benefits and risks of ERT have been widely debated. ERT has been shown to reduce the risk of cardiovascular disease (CVD), osteoporosis, stroke, and Alzheimer’s disease. However, the use of ERT has side effects, such as endometrial disease, breast cancer, vaginal bleeding, somatic complaints, and idiosyncratic reactions including hypertension and thrombosis [11]. The ideal treatment strategy for osteoporosis is to inhibit bone resorption by osteoclasts and/or increase bone formation by osteoblasts. Recently, interest in the treatment of osteoporosis with traditional Chinese medicines (TCM) is increasing, because they have fewer adverse reactions and are more suitable for long-term use [12]. Therefore, the chemical or natural compounds in TCM are a candidate for the treatment of metabolic bone disorders characterized by excessive osteoclastic bone resorption without side effects. As a representative example, genistein as botanical isoflavone are contained at relatively high concentrations in soybean. Genistein has suppressive effects on the osteoclastic differentiation of preosteoclastic RAW 264.7 cells in vitro [13]. Furthermore, genistein inhibits the bone loss induced by ovariectomized mice [14] and improved the quality of life and depression symptoms in osteopenic postmenopausal women [15,16].

Carotenoids are primarily produced within phytoplankton, algae and plants, and these pigments are responsible for a variety of colors seen in nature. The carotenoid pigment, astaxanthin, is found in salmon, trout and other aquatic animals [17]. Astaxanthin is not synthesized in animals and should be ingested as food [18]. Astaxanthin has a variety of biological activities, such as potent antioxidant effects both in vitro and in vivo [19], protective effects on asthma [20], therapeutic effect on ischemia-reperfusion injury [21], protective effect against liver damage [22], inhibitory effect on the proliferation of A549 lung cancer cells [23], and suppressive effect on neuroinflammation [24]. Recently, the oral administration of astaxanthin in the experimental periodontitis model was found to decrease the osteoclast number and increase the osteoblast number in the right mandibles [25]. However, the effect of astaxanthin on osteoclast differentiation and bone loss in osteoporosis animal models has not been studied yet.

Despite the existence of many reports concerning the biological effects of astaxanthin, no examination of the antiosteoporotic effects of astaxanthin on postmenopausal osteoporosis induced by ovariectomy in mice has been conducted to date. We hypothesized that astaxanthin would inhibit bone loss by inhibiting the activity of the osteoclasts. In the present study, we demonstrated the inhibitory effects of astaxanthin on osteoclast differentiation and trabecular bone loss.

## 2. Results

### 2.1. Effects of AST on Osteoclast Differentiation

**Figure 1:**
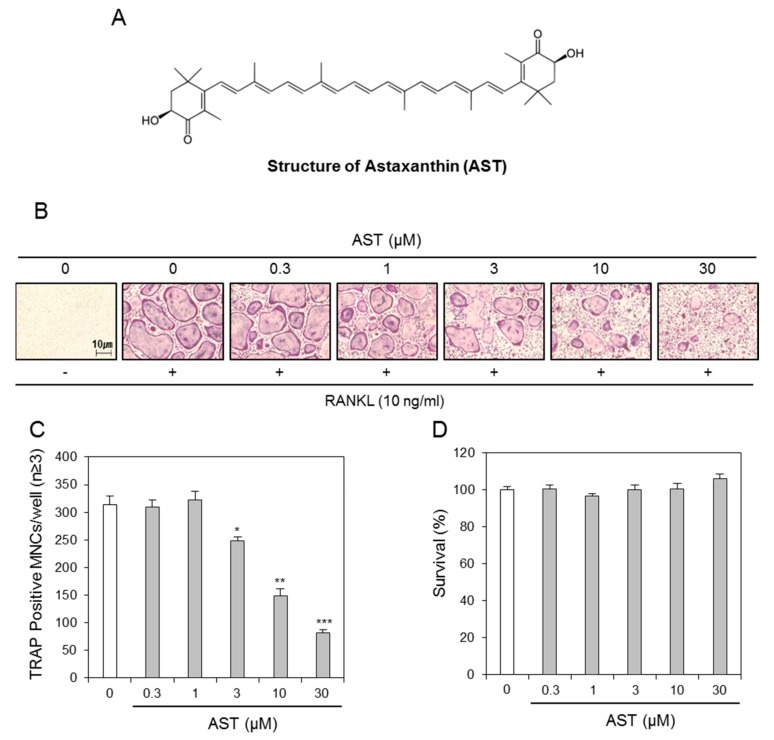
Astaxanthin suppresses osteoclastogenesis. (**A**) Chemical structure of Astaxanthin; (**B**) BMMs prepared from bone marrow cells were cultured for 4 days with RANKL (10 ng/mL) and M-CSF (30 ng/mL) in the presence of the indicated concentrations of Astaxanthin or 0.1% DMSO (control vehicle). The cells were fixed in 3.7% formalin, permeabilized in 0.1% Triton X-100, and stained for TRAP, a marker enzyme of osteoclasts; (**C**) TRAP-positive multinuclear cells (nuclei ≥ 3) were counted as osteoclasts. * *p* <0.05, ** *p* < 0.01, *** *p* <0.001; (**D**) The effect of Astaxanthin on the viability of BMMs was evaluated by CCK-8 assay. In (**C**, **D**), *n* = 3.

In order to evaluate the effect of AST (Figure 1A) on osteoclast differentiation, we added AST during osteoclast differentiation with RANKL and M-CSF. The treatment with AST was found to inhibit the osteoclast differentiation (Figure 1B) and the number of TRAP-positive multinucleated cells (Figure 1C). Furthermore, we investigated the cytotoxic effect of AST and found that it did not have any cytotoxic effects at any of the concentrations (Figure 1D). These results indicated that AST inhibits osteoclast differentiation without cytotoxicity.

### 2.2. Effects of AST on RANKL-Induced mRNA Expression of Osteoclast-Specific Genes

**Figure 2:**
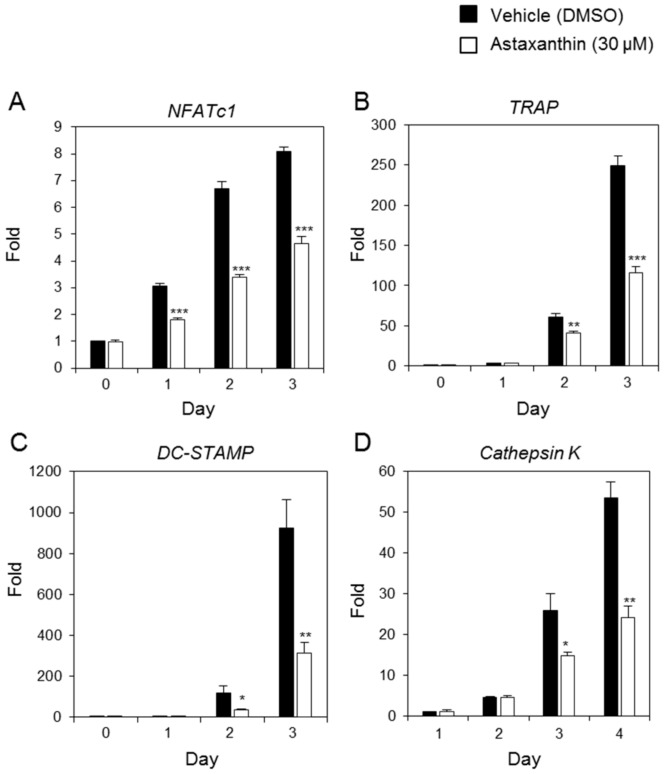
Astaxanthin inhibits the expression of the genes involved in osteoclastogenesis. BMMs were treated with 0.1% DMSO or Astaxanthin (30 μM) and then stimulated with RANKL (10 ng/mL) and M-CSF (30 ng/ml) for the indicated number of days. The expressed mRNA levels of (**A**) *NFATc1*, (**B**) *TRAP*, (**C**) *DC-STAMP*, and (**D**) *cathepsin K* were analyzed by real-time PCR compared with the DMSO control. * *p* <0.05, ** *p* <0.01, *** *p* <0.001 (*n* = 3).

**Figure 3:**
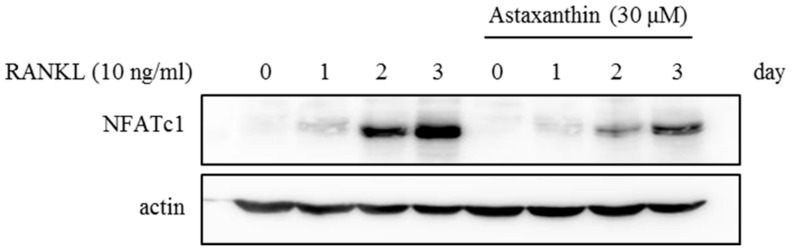
BMMs were pretreated with 0.1%DMSO or Astaxanthin (30 μM) for 1 h and then stimulated with RANKL (10 ng/mL) and M-CSF (30 ng/mL) for the indicated time. Cell lysates were resolved by SDS-PAGE, and western blotting was performed with anti-NFATc1 and actin antibodies as indicated.

To investigate effects of AST on the mRNA expression of osteoclast-specific genes, we performed real-time PCR. The mRNA expression levels of *NFATc1* (Figure 2A), *TRAP* (Figure 2B), *DC-STAMP* (Figure 2C), and *cathepsin K* (Figure 2D) were decreased by AST exposure compared with the vehicle. Additionally, the protein expression of *NFATc1* was significantly decreased by the AST treatment compared to the groups without AST (Figure 3). These results suggest that AST suppresses osteoclast formation through the expression of osteoclast-specific genes such as *NFATc1*, *TRAP*, *DC-STAMP*, and *cathepsin K*.

### 2.3. Effects of AST on Body Weight and Uterus Weight in Osteoporotic Mice

**Figure 4:**
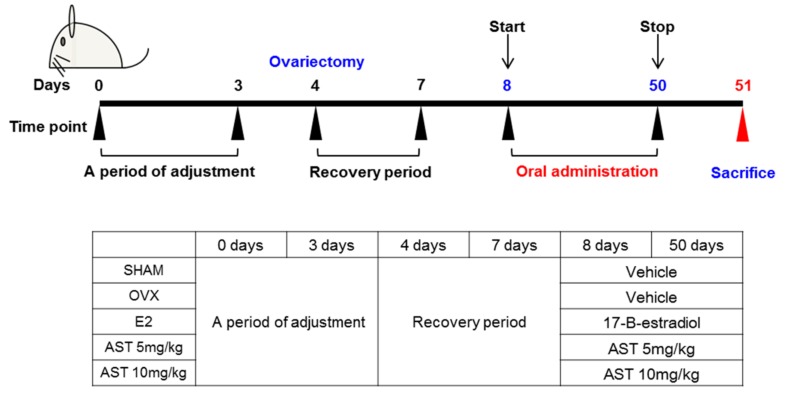
Experimental protocol for the induction and therapy of osteoporosis along with the treatment scheme.

**Figure 5:**
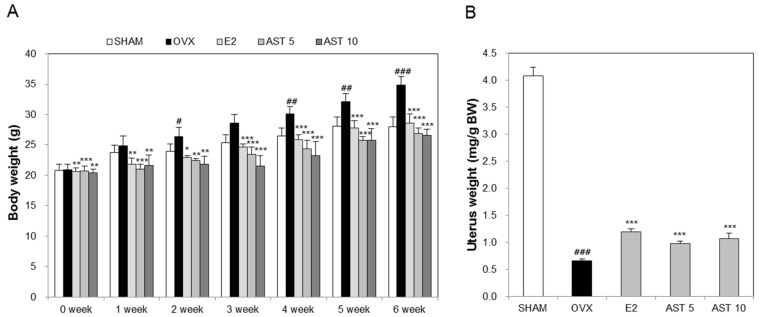
Effect of AST on body weight and uterus weight. The (**A**) body weight and (**B**) uterus weight measured at 24 h after the last treatment. Each value represents the mean ± SD for *n* = 5. # *p* < 0.05, ## *p* < 0.01, and ### *p* < 0.001 SHAM group vs. OVX group. * *p* < 0.05, ** *p* < 0.01, and *** *p* < 0.001 OVX group vs. E2, AST 5, and AST 10 group.

We conducted animal experiments using AST (Figure 4). To confirm the changes of the body weight during the 6-week period, we measured the body weight at weekly intervals. It was found that there was no difference in the initial body weight between the SHAM (20.8 ± 0.96 g) and OVX (21.20 ± 1.28 g) groups. The body weight of the E2 group (21.13 ± 0.57 g) and AST 10 group (20.91 ± 0.87 g) was decreased in comparison to that of the OVX group. The body weight of the AST 5 group (21.49 ± 0.47 g) was increased in comparison with that of the OVX group. The initial weight change was not large. However, the final body weight of the OVX group (34.82 ± 1.43 g) was significantly enhanced compared with that of the SHAM group (28.02 ± 1.56 g). The final body weight in the ovariectomy-induced mice was reduced by the oral administration of 5 mg/kg (26.89 ± 0.9 g) and 10 mg/kg (26.57 ± 0.99 g) of AST (Figure 5A). These results indicate that AST inhibits the increase in the body weight in the OVX mice.

In order to determine the effect of AST on the uterus weight in the OVX mice, we administered AST for 6 weeks and then weighted the uterus. The uterus weight of the OVX group was decreased compared to that of the SHAM group. However, the administration of E2, 5 mg/kg of AST, and 10 mg/kg of AST significantly suppressed the reduction of the uterus weight in the OVX mice (Figure 5B). The results suggest that AST contributes to the inhibition of the decrease in the uterus weight.

### 2.4. Effects of AST on Biochemical Markers in Osteoporotic Mice

**Figure 6:**
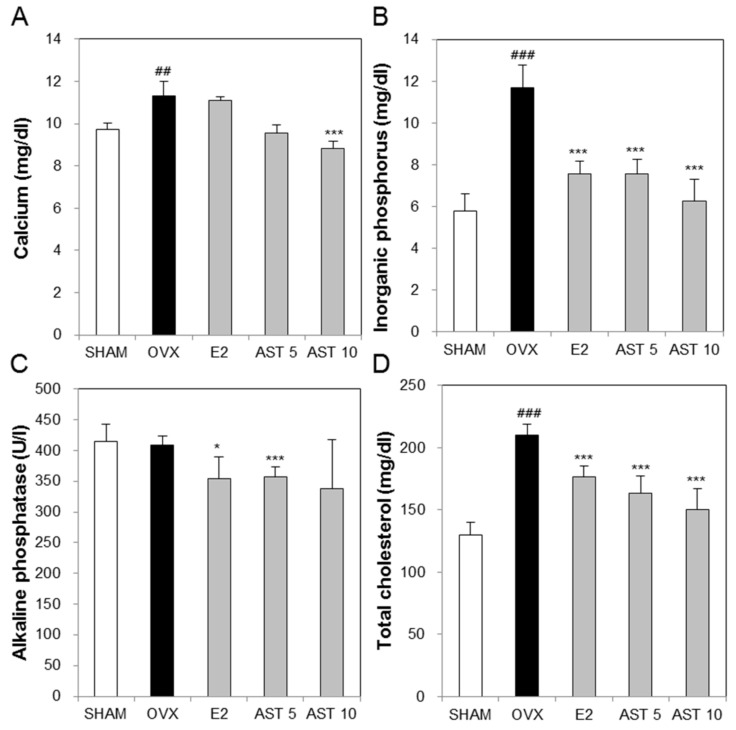
Effect of AST on serum biochemical markers. In the control, SHAM-operated mice and OVX mice with or without the administration of AST (5 and 10 mg/kg/day, p.o.) for 6 weeks, the serum (**A**) calcium, (**B**) phosphorus, (**C**) alkaline phosphatase, and (**D**) total cholesterol were determined by using a diagnostic slide. Each value represents the mean ± SD for *n* = 5. ## *p* < 0.01 and ### *p* < 0.001 SHAM group vs. OVX group. * *p* < 0.05 and *** *p* < 0.001 OVX group vs. E2, AST 5, and AST 10 group.

To evaluate the effect of AST administration on the biochemical markers in the osteoporotic mice, we measured the levels of calcium (Ca), inorganic phosphorus (IP), alkaline phosphatase (ALP), and total cholesterol (TCHO) in the serum. The Ca level of the OVX group was increased compared to that of the SHAM group. The increased Ca level in the OVX group was reduced by the oral administration of 10 mg/kg of AST (Figure 6A). Furthermore, the levels in the IP and TCHO groups were enhanced compared with those of the SHAM group. The exposure of the OVX mice to 5 mg/kg and 10 mg/kg of AST suppressed the enhancement of the levels in the IP and TCHO groups compared with those of the OVX group (Figure 6B,D). There was no difference in the ALP level between the SHAM and OVX groups. However, the administration of AST decreased the level of ALP (Figure 6C). These results indicate that the oral administration of AST reduces the enhancement of the biochemical parameters.

### 2.5. Effects of AST on the Level of TRAP in Osteoporotic Mice

**Figure 7:**
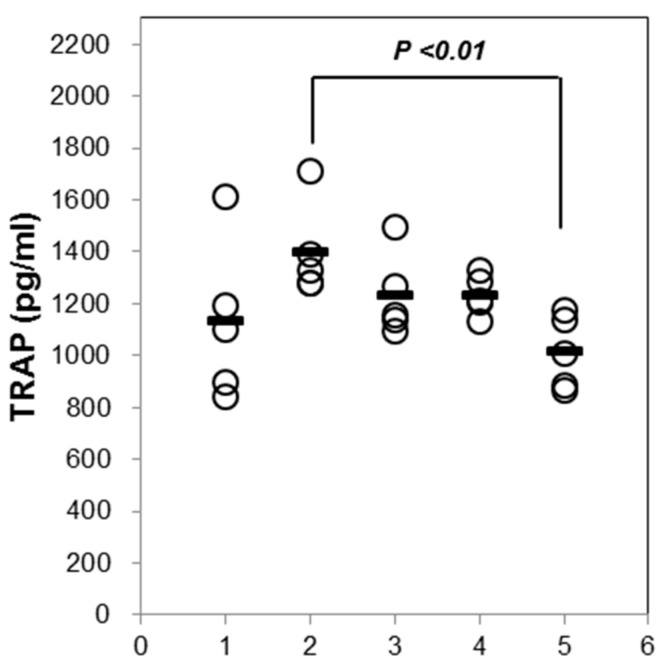
Effect of AST on serum tartrate-resistant acid phosphatase (TRAP) in the control, SHAM-operated mice and OVX mice with or without the administration of AST (5 and 10 mg/kg/day, p.o.) for 6 weeks. Serum TRAP was measured by ELISA kit. Each value represents the mean ± SD for *n* = 5. Group numbers (1 = SHAM, 2 = OVX, 3 = AST 5 mg/kg, 4 = 10 mg/kg).

To determine the effect of AST on the bone turnover makers, we measured the tartrate-resistant acid phosphatase (TRAP) in the serum. The serum TRAP activity in the OVX group was increased compared to that in the SHAM group. There was no significant difference between the SHAM group and OVX group. The exposure of the OVX mice to 10 mg/kg of AST decreased the TRAP activity (Figure 7). These results suggest that AST inhibited the TRAP activity.

### 2.6. Effect of AST on the Micro-Architecture of Proximal Tibia and Digital Femur

**Figure 8:**
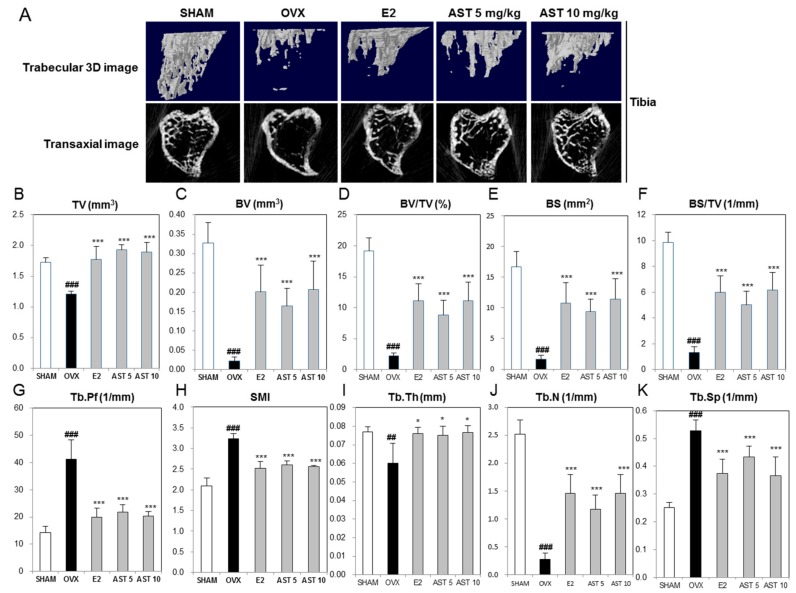
Effect of AST on trabecular morphometric parameters in proximal tibia of C3H/HeN mice. The mice were treated with the vehicle, AST (5 and 10 mg/kg/day, p.o.) for 6 weeks. (**A**) The three-dimensional micro-computed tomography images were analyzed by CTvol. (**B**) Tissue volume (TV), (**C**) bone volume (BV), (**D**) bone volume/tissue volume, (**E**) bone surface, (**F**) bone surface/tissue volume, (**G**) trabecular pattern factor, (**H**) structure model index, (**I**) trabecular thickness, (**J**) trabecular number, and (K) trabecular separation as analyzed with micro-CT Skyscan CTAn software. Each value represents the mean ± SD for *n* = 5. ## *p* < 0.01 and ### *p* < 0.001 SHAM group vs. OVX group. * *p* < 0.05 and *** *p* < 0.001 OVX group vs. E2, AST 5, and AST 10 group.

**Figure 9:**
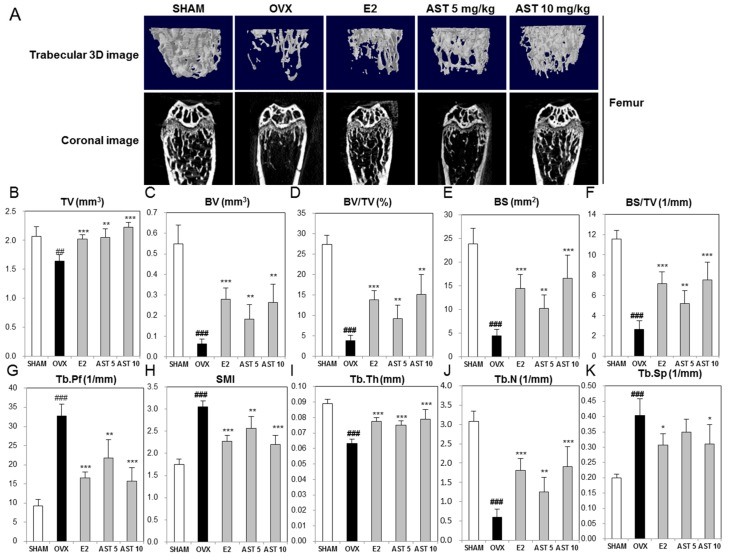
Effect of AST on trabecular morphometric parameters in distal femur of C3H/HeN mice. The mice were treated with the vehicle, AST (5 and 10 mg/kg/day, p.o.) for 6 weeks. (**A**) The three-dimensional micro-computed tomography images were analyzed by CTvol. (**B**) Tissue volume (TV), (**C**) bone volume (BV), (**D**) bone volume/tissue volume, (**E**) bone surface, (**F**) bone surface/tissue volume, (**G**) trabecular pattern factor, (**H**) structure model index, (**I**) trabecular thickness, (**J**) trabecular number, and (**K**) trabecular separation as analyzed with micro-CT Skyscan CTAn software. Each value represents the mean ± SD for *n* = 5. ## *p* < 0.01 and ### *p* < 0.001 SHAM group vs. OVX group. * *p* < 0.05, ** *p* < 0.01, and *** *p* < 0.001 OVX group vs. E2, AST 5, and AST 10 group.

To determine the effect of AST on the morphological changes of the tibia and femur in the OVX mice, we analyzed the micro-architecture in the trabecular bone by micro-computed tomography (micro-CT). The trabecular bone of the proximal tibia in the OVX group was decreased compared to that of the SHAM group. However, the exposure to AST increased the trabecular bone (Figure 8A). When compared with the SHAM group, the tissue volume (TV), bone volume (BV), BV/TV, bone surface (BS), BS/TV, trabecular thickness (Tb.Th), and trabecular number (Tb.N) of the OVX group were significantly decreased. The oral administration of AST (5 and 10 mg/kg/days, p.o.) enhanced the TV, BV, BV/TV, BS, BS/TV, Tb.Th, and Tb.N (Figure 8B–J). The trabecular pattern factor (Tb.Pf), structure model index (SMI), and trabecular bone separation (Tb.Sp) of the OVX group were increased compared with those of the SHAM group. The treatment with AST reduced the Tb.Pf, SMI, and Tb.Sp (Figure 8G,H,K). Similar to the microstructure of the proximal tibia, the oral administration of AST increased the trabecular bone in femur, TV, BV, BV/TV, BS, Tb.Th, and Tb.N, and decreased the Tb.Pf, SMI, and Tb.Sp (Figure 9). The analysis of the properties of the trabecular bone indicated that AST induces damage to the trabecular bone micro-architecture in OVX mice.

### 2.7. Effect of AST on Bone Mineral Density in Osteoporotic Mice

**Figure 10:**
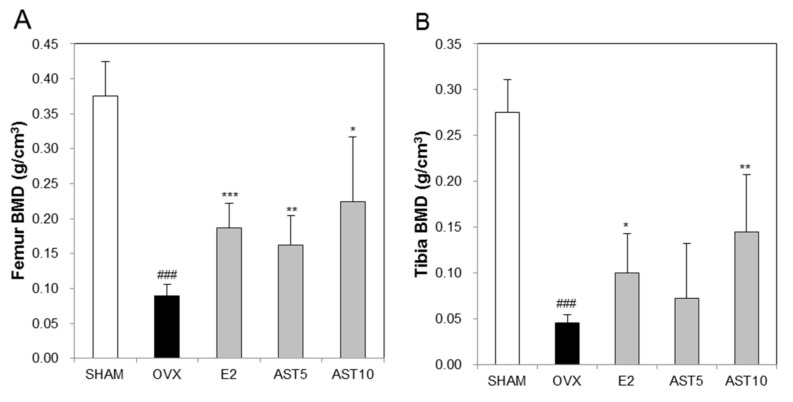
Effect of AST on bone mineral density (BMD) of trabecular in distal femur and proximal tibia of C3H/HeN mice in the control, SHAM-operated mice and OVX mice with or without the administration of AST (5 and 10 mg/kg/day, p.o.) for 6 weeks. (**A**) The femur BMD and (**B**) tibia BMD were analyzed by CTAn software. ### *p* < 0.001 SHAM group vs. OVX group. * *p* < 0.05, ** *p* < 0.01, and *** *p* < 0.001 OVX group vs. E2, AST 5, and AST 10 group.

In order to confirm the inhibitory effect of AST on the bone loss in the OVX mice, we quantified the trabecular bone mineral density (BMD) in the femur and tibia. The BMD of the OVX group was significantly reduced compared to that of the SHAM group. However, the oral administration of AST significantly suppressed the reduction of the BMD in the femur (Figure 10A) and tibia (Figure 10B). These results indicate that AST has an inhibitory effect on the cancellous bone loss.

### 2.8. Effect of AST on Histological Changes in Osteoporotic Mice

**Figure 11:**
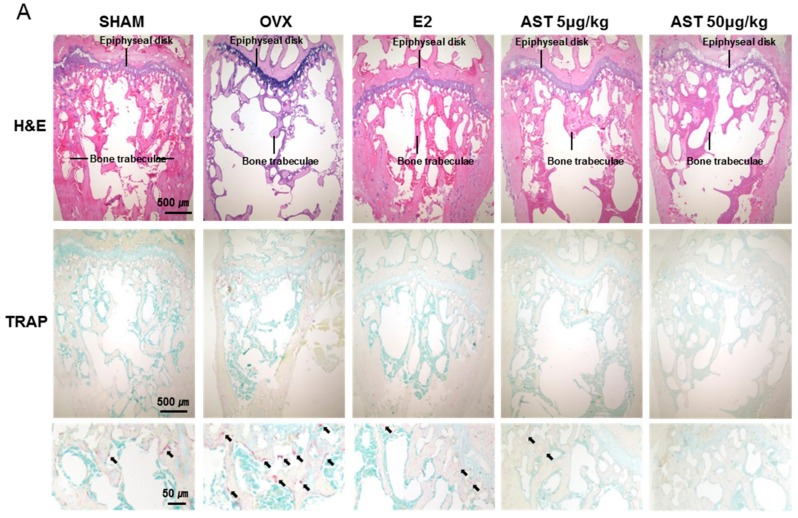

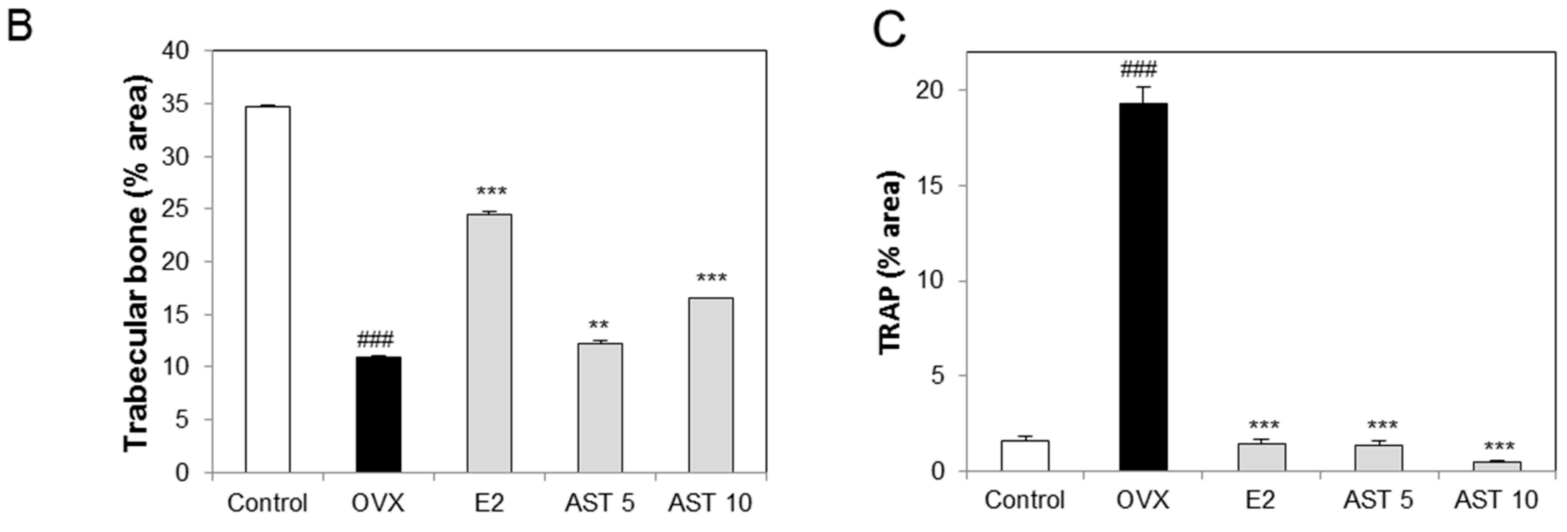
Effect of AST on bone tissue of trabecular in distal femur of C3H/HeN mice. The mice were treated with the vehicle, AST (5 and 10 mg/kg/day, p.o.), for 6 weeks. (**A**) Histological analysis of distal femur with hematoxylin and eosin (H&E) and tartrate-resistant acid phosphatase (TRAP) staining; (**B**) Trabecular and (**C**) TRAP positive cells in femur were analyzed by Image J program. Each value represents the mean ± SD for *n* = 3. ### *p* < 0.001 SHAM group vs. OVX group. ** *p* < 0.01 and *** *p* < 0.001 OVX group vs. E2, AST 5, and AST 10 group.

The digital femur of the mice in all of the groups underwent H&E staining to facilitate the observation of histological changes (Figure 11A). The trabecular bone of the OVX group was decreased compared with that of the SHAM group. However, the trabecular bone area in the AST oral administration group (AST 5 and 10 mg/kg) was enhanced compared to that of the OVX group (Figure 11B). The TRAP positive cells numbers of the OVX group were increased compared to those of the SHAM group. The treatment of the OVX mice with AST suppressed the TRAP activity (Figure 11C). These results suggest that AST has inhibitory effects on the TRAP activity.

## 3. Discussion

An estimated ten million Americans have osteoporosis, and over 1.5 million fractures per year are attributed to osteoporosis, including approximately 300,000 hip fractures. Especially, the mortality rate of hip fracture is high. Osteoporosis and osteoporotic fractures reduce quality of life, and the cost of osteoporosis is an increasingly significant public health concern [26].

Most adult skeletal diseases, such as osteoporosis, periodontal disease, rheumatoid arthritis, multiple myeloma and metastatic cancers, are due to excess osteoclastic activity. Osteoclasts are created by the differentiation of monocyte/macrophage precursors cells at the bone surface. In the development of osteoclasts, macrophage colony stimulating factor (M-CSF) and the receptor activator of nuclear factor kappa-B ligand (RANKL) are required to induce the expression of genes that typify the osteoclast lineage, including those encoding tartrate-resistant acid phosphatase (TRAP) [27]. The synthesis of TRAP was found to be significantly greater in osteoclast-like cells and it exhibited osteolytic activity. In the present study, we confirmed that astaxanthin (AST) significantly inhibits the formation of osteoclasts from bone marrow-derived macrophages (BMMs) treated with RANKL and M-CSF, but had no cytotoxic effect on BMMs.

RANKL activates the nuclear factor of activated T cells (NFAT) c1, which induces a number of genes involved in cell differentiation [7]. NFATc1 induces the expression of fusion-mediating molecules, such as dendritic cell-specific transmembrane protein (DC-STAMP) [28]. Moreover, TRAP and the cysteine protease, cathepsin K, are among the downstream targets of NFATc1 [29]. Therefore, in order to determine the mechanism by which AST suppresses osteoclast differentiation, we confirmed the effect of AST on the mRNA levels of NFATc1, TRAP, DC-STAMP, and cathepsin K. The expression of NFATc1, TRAP, DC-STAMP, and cathepsin K mRNA was decreased by AST treatment in BMMs treated with RANKL. Moreover, AST suppressed the expression of NFATc1 protein. These results suggest that AST inhibits the formation of osteoclasts by suppressing the expression of NFATc1 in the M-CSF and RANKL-induced osteoclastogenesis pathway.

Various animal species, such as rodents, rabbits, dogs, and primates, have been used as experimental animal models in osteoporosis. Among these, the laboratory rat is the most appropriate for osteoporosis research, because the animals used must comply with national and local ethical and legislative considerations, be easy and safe to handle, have a low cost of acquisition, and require little maintenance [30]. Recently, osteoporosis research using mice instead of rats has been carried out [31,32,33,34]. In the present study, we demonstrated the protective effects of AST on osteoporosis using the ovariectomized (OVX) mice model.

In the in vivo study, the deficiency of estrogen increased the body weight and decreased the uterus weight. Especially, atrophy of organs such as the uterus is evidence of the success of ovariectomy [35]. In addition, the enhancement of the body weight in OVX mice is due to fat body fat accumulation caused by estrogen deficiency [36]. In the present study, we showed that the enhancement of the body weight and atrophy of the uterus in OVX mice, which was also observed in other studies, was limited by the treatment with 17β-estradiol [37,38,39,40]. In the present study, the oral administration of AST for 6 weeks inhibited the increase in the body weight and atrophy of the uterus.

Calcium, as an essential nutrient, is involved in most metabolic processes and most body calcium (99%) is located in the skeleton. The large amount of calcium in the skeleton is the source of its mechanical strength. Established osteoporosis is commonly associated with a negative calcium balance caused by problems such as the malabsorption of calcium and/or high obligatory calcium excretion [41]. Previous research showed that estrogen treatment increases calcium absorption in postmenopausal osteoporosis [42]. Phosphate is one of the minerals in the body and maintaining phosphate balance is of biological importance for bone health. Inorganic phosphorus is one of the main ionic components required for hydroxyapatite formation during the mineralization of the extracellular matrix [43]. Some authors reported that phosphorus levels in estrogen deficient mice were decreased in comparison to the sham group [44,45]. Moreover, one study reported that the total cholesterol concentration in postmenopausal women was significant increased [46]. The total cholesterol is an osteoporotic fracture risk factor. High total cholesterol in serum is a long-term cause of osteoporotic fracture [47]. In the experimental animal model produced by ovariectomy, the serum total cholesterol was increased compared to the intact mice group [48,49]. The ALP level, which is well known as a biochemical marker, has been reported to be elevated in the serum of ovariectomized animals [50]. In the present study, we showed that the levels of calcium, phosphorus, and total cholesterol, which are reduced in OVX mice, were increased by their exposure to AST.

Osteoporosis is defined as the impairment of bone architecture. As new products and methods have been developed for therapy, effective and sensitive non-invasive means able to detect early changes in the bone fragility process have also been developed. Knowledge of the bone microarchitecture provides a clue to improving its diagnosis and treatment. The parameters of the bone microarchitecture were assessed via high-resolution computed tomography (CT), micro CT, high-resolution magnetic resonance (MR) and micro MR [51]. Bone histomorphometry is performed to obtain quantitative information on bone remodeling and structure. These parameters provide information about bone mass, are calculated from measurements of the total bone area and are related to the bone strength [52]. Histomorphometric parameters include bone volume/tissue volume (BV/TV), trabecular thickness (Tb.Th), trabecular number (Tb.N), trabecular separation (Tb.Sp), structure model index (SMI), and trabecular pattern factor (Tb.Pf) [53,54]. Previous studies showed that BV/TV, Tb.Th, and Tb.N were decreased and Tb.Sp, SMI, and Tb.Pf were increased in OVX mice [55,56]. In this study, we showed that the oral administration of AST increased the TV, BV, BV/TV, BS, BS/TV, Tb.Th, and Tb.N and decreased the Tb.Pf, SMI, and Tb.Sp in the tibia and femur. Also, this study evaluated the effects of AST on the histological status of the femoral bone via hematoxylin and eosin (HE) and TRAP staining. We demonstrated that the oral administration of AST in OVX mice inhibits the trabecular bone loss by suppressing the increase in the osteoclast activity. These results indicate that the exposure of OVX mice to AST suppresses the destruction of the bone architecture.

The emergence of bone mass measurements to quantify the risk of bone deterioration has led to the development of the field of osteoporosis. Bone mass is the most important determinant of bone strength [57]. This study demonstrated that the administration of AST inhibited the reduction of BMD levels in animal models of osteoporosis. These results show that AST has potential as a therapeutic agent for osteoporosis.

In the present study, we demonstrated the inhibitory effects of AST on osteoclast formation in primary precursor cells. In the in vitro experiment, we showed that the inhibitory effect of AST on osteoclast formation is due to the inhibition of NFATc1 and DC-STAMP expression. Furthermore, the oral administration of AST in OVX-induced osteoporotic mice reduced osteoclast activity, which lowered the BMD level and inhibited the destruction of the bone microarchitecture. From a therapeutic point of view, AST is considered a good candidate for the preservation of bone loss in postmenopausal osteoporosis via inhibition of osteoclast differentiation and reduction of uterus weight.

## 4. Materials and Methods

### 4.1. Reagents

Astaxanthin (3,3′-dihydroxy-β-carotene-4,4′-dione; AST; Figure 1A) and dimethyl sulfoxide (DMSO) were purchased from Sigma-Aldrich Co. (St. Louis, MO, USA).

### 4.2. Cell Cultures and Osteoclast Differentiation

Bone marrow cells (BMCs) were isolated from the femur and tibia of 5-week-old male ICR mice (*n* = 2: Damool Science, Daejeon, Korea) by flushing with α-minimum essential medium (α-MEM; Invitrogen Life Technologies, Carlsbad, CA, USA) containing 100 units/mL penicillin and 100 µg/mL streptomycin (Invitrogen, Carlsbad, CA, USA). The BMCs were cultured with M-CSF (10 ng/mL) for 1 day, and the nonadherent cells were cultured for a further 3 days in the presence of M-CSF (30 ng/mL). Bone marrow macrophages (BMMs) were obtained from BMCs cultured on a petri dish in α-MEM supplemented with 10% fetal bovine serum (FBS; Invitrogen Life Technologies, CA, USA) with 30 ng/mL of mouse recombinant macrophage colony-stimulating factor (M-CSF; PEPROTECH, Rocky Hill, NJ, USA) for 3 days. The BMMs were seeded and cultured in the presence of 10 ng/mL of mouse recombinant receptor activator of nuclear factor-κB ligand (RANKL; R&D Systems, Minneapolis, MN, USA) and 30 ng/mL of M-CSF for 4 days in the presence or absence of astaxanthin.

### 4.3. Cytotoxicity Assay for Astaxanthin

BMMs were seeded in 96-well plates (1 × 10^4^ cells/well) with M-CSF (30 ng/mL) and astaxanthin. After incubation for 3 days, the cell viability was measured by using a CCK-8 kit (Dojindo Molecular Technologies, Kumamoto, Japan) according to the manufacturer’s protocol.

### 4.4. Tartrate-Resistant acid Phosphatase (TRAP) Staining Assay

After incubation with differentiation media containing astaxanthin/RANKL, the cultured cells were fixed with 3.7% formalin for 5 min and washed with distilled water. The fixed cells were permeabilized with 0.1% Triton X-100 for 10 min and stained with TRAP solution (Sigma-Aldrich) for 10 min. The TRAP positive multinucleated cells (cells with 3 nuclei or more; nuclei ≥ 3) were counted as mature osteoclasts.

### 4.5. Real-Time PCR

Real-time PCR was performed as described previously [58]. The primers for real-time PCR were designed (Table 1) by using the Primer3 design program [59]. Quantitative-PCR was completed by using a real-time PCR detection system (Bio-Rad, Hercules, CA, USA) and TOPreal qPCR 2× PreMIX (Enzynomics, Daejeon, Korea). All tests were run in triplicate, and the data were analyzed by the 2^−ΔΔCt^ method.

### 4.6. Western Blot Analysis

Western blotting was performed as described previously [9]. The cells were washed with phosphate-buffer saline (PBS) and lysed including the 1 mM phenylmethylsulfonyl fluoride (PMSF; Bio Basic, Ontario, CA, USA) and 5 μg/mL leupeptin (Sigma-Aldrich). The cell lysates were isolated by centrifugation at 15,000 rpm for 12 min. The proteins (20 μg) were subjected to 10% sodium dodecyl sulfate-polyacrylamide gel electrophoresis (SDS-PAGE) and then transferred onto a polyvinylidene difluoride (PVDF) membrane (Amersham Biosciences, Little Chalfont, NJ, USA). The membrane was blocked by 5% skim milk and then incubated overnight at 4 °C with a primary antibody, as indicated. The membrane was washed and then incubated with horseradish peroxidase (HRP)-conjugated secondary antibody. The protein bands were visualized by using MicroChemi 4.2 (DNR Bio-imaging System, Jerusalem, IL, USA) and Super-Signal West Pico Chemiluminescent Substrate (Pierce Chemical, Rockford, IL, USA).

### 4.7. Experimental Animals

C3H/HeN female mice (eight weeks of age, weight 21 ± 1 g) were obtained from Orientbio (Orientbio Inc., Iksan, Korea). The mice were housed in standard polycarbonate cages under controlled conditions at 22 ± 2 °C in 50 ± 5% humidity under a 12-h light/dark cycle. The commercial rodent chow (DAE-HAN Biolink, Daejeon, Korea) and water were provided during the experimental period (6 weeks). Prior to the experiments, the mice were allowed to adapt to the laboratory environment for 3 days. The animals were anesthetized using Zoletil and Rumpun induced anesthesia prior to undergoing surgery. Sham operations were performed by exteriorizing the ovaries, and bilateral OVX procedures were performed using the dorsal approach. The animals were allowed to recover from surgery for 3 days prior to the experiments. After surgery, the mice were divided into two groups; the sham group (*n* = 5) and ovariectomized (OVX, *n* = 25) group. The OVX mice were then randomly divided into four groups; the OVX group (*n* = 5), OVX with estradiol group (E2, *n* = 5, 0.03 μg/head/day), OVX with AST 5 group (*n* = 5, given 5 mg/kg/day) and AST 10 group (*n* = 5, given 10 mg/kg/day). E2 treatment was used as a positive control to compare the effect of AST on osteoporosis. The vehicle, E2, and AST were administered for 6 weeks, and the body weights were recorded weekly. After administration of the vehicle, E2, and AST, blood and various tissues from the sacrificed animals were collected for biochemical and morphological analysis. Sera were collected and stored at −80 °C until use, and the uteruses, tibias and femurs were removed and weighed. All mice were treated in strict accordance with the Sunchon National University Institutional Animal Care and Use Committee’s (SCNU IACUC) guidelines for the care and use of laboratory animals. All procedures were approved by the SCNU IACUC (permit number: SCNUIACUC-2017-06, approval’s date: 22 May 2017).

### 4.8. Measurements of Serum Ca, IP, ALP, and TCHO

The serum was obtained by centrifugation of blood at 5000 rpm for 5 min. The serum was immediately stored at −80 °C. The serum calcium (Ca), inorganic phosphorus (IP), alkaline phosphatase (ALP), and total cholesterol (TCHO) levels were were detected using the o-cresolphthalein complexone (CPC) method and an automated blood analyzer (Dri-Chem 3500i; Fujifilm Medical System Co., Ltd., Tokyo, Japan).

### 4.9. Measurements of TRAP and BALP in Serum by ELISA

The tartrate-resistant acid phosphatase (TRAP) activity (a marker of bone resorption) and the bone alkaline phosphatase (BALP) levels were measured using a TRAP enzyme-linked immunoassay (ELISA) kit (USCN Life Science, Wuhan, China) and a BALP ELISA kit (Elabscience, Wuhan, China), respectively.

#### 4.1.10. Analysis of Trabecular Bone Morphometric Parameters

The structural properties on the trabecular bone of the proximal tibia and distal femur were determined by a high-resolution micro-computed tomography (micro-CT) system (SkyScan 1272, Bruker micro-CT, Kontich, Belgium). The bones were covered with dental wax to prevent movement during the scanning. The scans were taken with a source voltage of 60 kV and a source current of 166 μA. The resolution was set to 13.265 μm and the rotation step was 0.2°. Image reconstruction was performed by NRecon software (1.1.9, SkyScan, Kontich, Belgium). The trabecular bone of the proximal tibia and distal femur was manually segmented from the cortical bone, and the trabecular bone parameters were analyzed over 100 slices from the slices without growth plate. A morphometric analysis was conducted to determine the three-dimensional (3D) bone structure in vivo. We obtained the bone morphometric parameters of the bone cleaned of adherent soft tissues, including the tissue volume (TV), bone volume (BV), bone volume/tissue volume (BV/TV), bone surface (BS), bone surface/tissue volume (BS/TV), trabecular thickness (Tb.Th), trabecular separation (Tb.Sp), and trabecular number (Tb.N). Two-dimensional (2D) and 3D images were obtained for visualization and display. The structural parameters for the trabecular bone were analyzed using CTAn software 1.1.9 (SkyScan, Kontich, Belgium). The 3D image of the trabecular bone was regenerated through CTvol software. For the quantification of the trabecular volumetric mineral density (BMD), the micro-CT was calibrated using two standard phantoms with densities of 0.25 and 0.75 g/cm^3^.

#### 4.1.11. Histological Analysis

The femur was fixed in 4% formaldehyde at room temperature and decalcified in 10% ethylenediaminetetraacetic acid (EDTA). The femur was dehydrated, embedded in paraffin, sectioned at 5 μm, and stained with hematoxylin and eosin (H & E). To measure the osteoclast activity in the bone tissue, we stained the bones with TRAP agents. For the TRAP staining of the femur, 225 μM naphthol AS-MX phosphate (Sigma-Aldrich), 0.84% *N*,*N*-dimethylformamide (Sigma-Aldrich), and 1.33 mM Fast Red Violet LB Salt (Sigma-Aldrich) in 50 mM sodium acetate (pH 5.0) containing 50 mM sodium tartrate were used. The reagent treated sections were washed in distilled water and counterstained with 1% methyl green. The image J program (National Institutes of Health, Bethesda, MD, USA) was used to analyze the trabecular bone and TRAP positive cells. The trabecular area (%) was measured as the ratio of the trabecular bone area to the total bone area. The measurement of TRAP positive cells (TRAP% area) was quantified relative to the total trabecular bone surface.

#### 4.1.12. Statistical Analysis

The results are presented as means ± SDs. Statistical analyses were performed using SPSS version 22 (SPSS, Chicago, IL, USA). The Student’s *t*-test was used to determine the significances of the differences between the groups. *p* Values of *p* < 0.05 were considered statistically significant.

## Figures and Tables

**Table 1:** Primer sequences used in this study.

Gene of Interest	Primer Sequence (5′→3′)	
	Sense	Anti-Sense
NFATc1	GGGTCAGTGTGACCGAAGAT	GGAAGTCAGAAGTGGGTGGA
cathepsin K	GGCCAACTCAAGAAGAAAAC	GTGCTTGCTTCCCTTCTGG
DC-STAMP	CCAAGGAGTCGTCCATGATT	GGCTGCTTTGATCGTTTCTC
TRAP	GATGACTTTGCCAGTCAGCA	ACATAGCCCACACCGTTCTC
GAPDH	AACTTTGGCATTGTGGAAGG	ACACATTGGGGGTAGGAACA

GAPDH: glyceraldehyde-3-phosphate dehydrogenase.
